# Interruption of Electrical Conductivity of Titanium Dental Implants Suggests a Path Towards Elimination Of Corrosion

**DOI:** 10.1371/journal.pone.0140393

**Published:** 2015-10-13

**Authors:** Alex E. Pozhitkov, Diane Daubert, Ashley Brochwicz Donimirski, Douglas Goodgion, Mikhail Y. Vagin, Brian G. Leroux, Colby M. Hunter, Thomas F. Flemmig, Peter A. Noble, James D. Bryers

**Affiliations:** 1 Department of Oral Health Sciences, University of Washington, Box 357444, Seattle, Washington, United States of America; 2 Department of Periodontics, University of Washington, Box 357444, Seattle, Washington, United States of America; 3 Department of Physics, Chemistry and Biology (IFM) Linköping University, SE-581 83, LINKÖPING, Sweden; 4 PhD Program in Microbiology, Alabama State University, Montgomery, Alabama, United States of America; 5 Faculty of Dentistry, The University of Hong Kong, Prince Philip Dental Hospital, 34 Hospital Road, Sai Ying Pun, Hong Kong SAR, Peoples’ Republic of China; 6 Department of Bioengineering, University of Washington, 3720 15th Avenue NE, Seattle, Washington, United States of America; University of Zaragoza, SPAIN

## Abstract

Peri-implantitis is an inflammatory disease that results in the destruction of soft tissue and bone around the implant. Titanium implant corrosion has been attributed to the implant failure and cytotoxic effects to the alveolar bone. We have documented the extent of titanium release into surrounding plaque in patients with and without peri-implantitis. An *in vitro* model was designed to represent the actual environment of an implant in a patient’s mouth. The model uses actual oral microbiota from a volunteer, allows monitoring electrochemical processes generated by biofilms growing on implants and permits control of biocorrosion electrical current. As determined by next generation DNA sequencing, microbial compositions in experiments with the *in vitro* model were comparable with the compositions found in patients with implants. It was determined that the electrical conductivity of titanium implants was the key factor responsible for the biocorrosion process. The interruption of the biocorrosion current resulted in a 4–5 fold reduction of corrosion. We propose a new design of dental implant that combines titanium in zero oxidation state for osseointegration and strength, interlaid with a nonconductive ceramic. In addition, we propose electrotherapy for manipulation of microbial biofilms and to induce bone healing in peri-implantitis patients.

## Introduction

Placement of dental implants is a common procedure for patients that lose a tooth, in order to restore function and aesthetics. While osseointegrated implants have been in commercial use since 1978 (according to Nobel Biocare), there are still problems related to their use. Issues such as peri-implantitis, possible titanium leaching, and corrosion of the titanium are among the issues associated with titanium implants [1].

Peri-implantitis is an inflammatory response that results in the destruction of soft tissue and bone around the implant [2]. It impairs oral health-related quality of life in affected patients and is a major reason for the failure of implant-supported dental prostheses. Approximately one out of four patients with dental implants develop peri-implantitis within 11 years following implant placement [3].

Peri-implantitis has been associated with history of periodontitis, bacterial plaque, bleeding, bone level on the medium third of the implant, poor prosthetic fit, suboptimal screw joint, metal-ceramic restorations and the interaction between bacterial plaque and the proximity of other teeth or implants [4]. More recently, high throughput DNA sequencing analysis has demonstrated that the microbial communities associated with peri-implantitis are distinct from peri-implant healthy communities and those found in periodontitis [5]. Nevertheless, according to a recent systematic review [6], there is no consensus about the etiology of peri-implantitis and its relation to periodontitis.

The potential role of titanium metal in peri-implantitis has recently received attention [7,8]. Titanium is a relatively active metal, with the standard electrochemical potential of –1.628V for the reaction Ti^2+^(aq) + 2e → Ti(s) (1atm, 25°C, 1M Ti^2+^). In sterile conditions, cytotoxic effects by titanium wear products have been reported for orthopedic implants. Discoloration, inflammation, and tissue necrosis have been observed around areas of corroded and worn titanium implants [8]. Similarly, an inflammatory tissue response has been found for titanium dental implants that were completely covered in soft tissue [7, 9].

Although a layer of titanium oxide (TiO_2_) protects the titanium surface from reacting with electron acceptors such as oxygen, the oxide layer, however, is not stable under acidic conditions. Microbial-mediated corrosion of titanium has been demonstrated *in vitro* [10]. Specifically, *Streptococcus mutans* biofilm grown on a titanium surface and exposed to artificial saliva decreased in pH and a thinning of the oxide layer was observed within 7 days of exposure. On a much shorter time scale, titanium corrosion products were detected after just 90 min of exposure to *S*. *mutans* [11]. In pooled samples of exfoliated cells from several human subjects, elevated levels of titanium were found at the sites with peri-implantitis but not at sites associated with health [12]. It is important to understand that the corrosion mediated by the microorganisms and their metabolites is not necessarily the only source of titanium that is leached into the surrounding tissues. Specifically, Addison et al [13] proposed that titanium may be released due to micro-motion and localized corrosion in surface crevices. The body itself is capable of corroding titanium aside from the wear and microorganisms [14].

Dental implants are exposed to concentration gradients of various chemicals in the oral cavity due to differential oxygen partial pressures in supramucosal and submucosal environments as well as gradients of numerous microbial metabolites. Spatial gradients in oxygen on metal implant can lead to cathodic half-cells and the generation of an electrical current. From the field of microbial fuel cells, it is known that a metal electrode is capable of directly providing electrons to bacteria in a biofilm (i.e., acting as a biocathode), which in turn could transfer electrons to oxygen, fumarate, and iron compounds. At the same time, bacteria are capable of transferring electrons to the electrode; hence, the electrode may function as a bioanode [15, 16]. We conjectured that due to oxygen and metabolite gradients in a patient’s mouth, a titanium dental implant will develop cathodic and anodic zones covered with corresponding biofilms. The biofilms will produce numerous substances, some of which will participate in the electrochemical processes. Because titanium is electrically conductive, it is natural to assume that an electric current would flow between the cathodic and anodic zones.

The goals of this research are two-fold: to quantify titanium corrosion and leakage occurring among the patients having implants and to simulate titanium implant corrosion in a bioreactor. We realize that the leached titanium ions will turn into either predominantly insoluble form or bind to biomolecules. Therefore our analytical technique will ensure digestion of such forms of titanium. In addition, it is clear that numerous biological substances are expected to present in a surface biofilm, which will affect the corrosion potential of Ti [17] making the modeling of implant corrosion very difficult. This is why we will use actual oral microbiota to inoculate our bioreactor.

We hypothesized that the electro-conductive property of titanium implants is responsible for the formation of a closed electrochemical circuit that causes titanium corrosion. If supported then it might be possible to develop corrosion-resistant implants that interrupt electrical conduction. To address the first goal, we will measure quantities of titanium in the subgingival plaque of healthy and peri-implantitis patients. To address the second goal, we will put titanium implants in an *in vitro* microbial reactor system containing oral cavity microbiota and create an electrochemical circuit through the implants that could be operated as closed or open.

## Materials and Methods

### Collection of dental plaque

#### Subject Recruitment

The Institutional Review Board at the University of Washington approved the study protocol and all patients provided written informed consent. Patients who had dental implants placed at the Graduate Periodontics Clinic, University of Washington, between 1998 and 2003 and who had radiographs taken after initial remodeling were recruited for this study. There were no exclusion criteria. Additional implants placed before or after the baseline were not included in the study. A prior publication provides details of the 96 patients with 225 implants regarding the conditions at time of placement including: the periodontal status of the patient, brands of implants, type of restoration, patient factors, and the subsequent clinical findings at follow-up exams [3]. Those findings include data on the prevalence of peri-implantitis and peri-implant mucositis, along with a predictive model for implant failure.

#### Plaque collection for titanium analysis

A subset of patients returned for an additional visit in August to December 2014, in which a microbial sample was taken from implants that had been diagnosed as either healthy or with peri-implantitis. Implants with a diagnosis of mucositis were not included. The sample was collected from the deepest probing site at each dental implant utilizing a sterile curette. The curette was carefully inserted to the base of the pocket with the blade facing the gingival side, not the implant surface, and plaque was removed in an upward motion facing the gingiva. This method was used to obtain an adequate volume of plaque for the titanium and microbial analysis on an individual implant basis. After supragingival plaque removal, the curette was inserted into the base of the pocket and plaque was collected from the base of the pocket to the gingival margin. The plaque was transferred to a screw-cap tube with 500μl of sterile water and frozen at -80°C for future processing. These patients were again given a periodontal diagnosis and the latest diagnosis was used in the analysis present in this report.

#### Definitions

For this study, peri-implant mucositis was defined as the presence of bleeding on probing and/or gingival inflammation with no evidence of radiographic bone loss beyond normal remodeling. Peri-implantitis was defined as the presence of bleeding on probing and/or suppuration, with 2 mm of detectable bone loss following initial remodeling, and a probing depth of 4 mm or greater. The presence of 2 mm of bone loss alone without mucositis symptoms did not count as a case of peri-implantitis. Due to non-standardized radiographs at the prosthetic insertion and follow-up examination, we included the case definition of a threshold of 2 mm from the expected marginal bone level following remodeling post-implant placement [18].

#### Determination of periodontal status

Full mouth periodontal chartings were utilized to assign a periodontal diagnosis. The diagnosis was assigned by the examiner (DD) as healthy, gingivitis, slight, moderate, or severe chronic periodontitis, with slight periodontitis defined as 1–2 mm of attachment loss and moderate and severe as 3–4 mm and 5 mm or more attachment loss respectively [19].

### *In vitro* experiments

#### Bacterial culture conditions

We used the same medium for all *in vitro* experiments, which was made of the autoclaved medium base supplemented with filter-sterilized vitamin K and hemin solutions [20]. Specifically, the base medium was composed of Trypticase Soy Broth 30 g/l and Yeast Extract 5 g/l, pH adjusted to 7.2 and autoclaved. Each 1L of medium base was supplemented with Vitamin K3 stock 0.2 ml and Hemin stock 10 ml. Hemin stock was prepared from 50 mg Hemin dissolved in 1 ml 1M NaOH subsequently diluted with 99 ml water. Hemin stock was stored at 4°C. Vitamin K3 stock was prepared by dissolving 250 mg Vitamin K in 50 ml 98% ethanol. Vitamin K3 stock was stored at 4°C.

#### Electrochemical measurements

**Fig 1:**
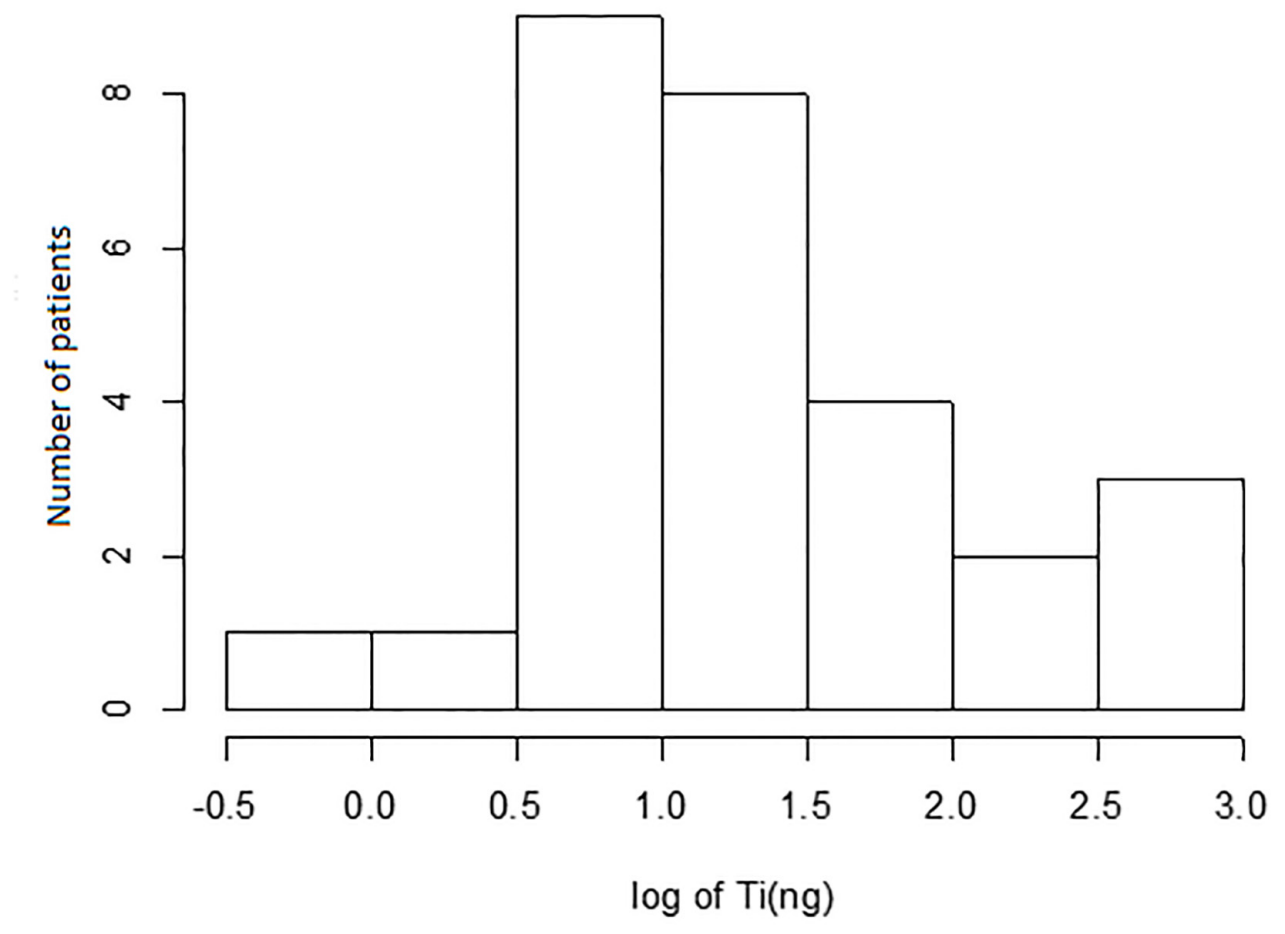
Electrochemical cell for investigation of titanium implants. a, diagram of the cell, b.—actual photograph of the setup, c—electrical schematic diagram.

The two-terminal symmetrical electrochemical cell (Fig 1) was made of a glass tube capped with rubber stoppers. The tube was made in two versions: with and without inlet/outlets. Two titanium dental implants, Straumann SLActive, length 14 mm, diameter 4.8 mm, have been integrated into the cell by a mechanical attachment to titanium wires inserted through the stoppers via sealed glass tubes. The top stopper was outfitted with two 16-gauge needles to enable inoculation and gas exchange. The cell was filled with medium under sterile conditions using compressed air, needles and sterilized hoses. The upper implant was immersed into the medium only halfway. After the electrochemical cell was filled up, it was placed into the thermostated chamber for at least 12 h to ensure no contamination occurred during the medium transfer.

The electrochemical cell was loaded with a resistor bank of ~0 kOhm, 10 kOhm, 100 kOhm, 1 MOhm values (Fig 1c). The resistor bank was housed in a grounded metal box that rested inside the 37°C thermostated chamber in order to maintain constant values of the resistors. Switching of the resistors in the resistor bank was performed by reed-switch relays that were energized from an electronic interface circuit receiving commands from the computer. The interface circuit was based on a microcontroller, which received an ASCII character from a PC and converted it to 8 bits across the output lines (S2 Fig). For the voltmeter, we used a VWR Symphony SP90M5 pH meter (input impedance greater than 10^13^ Ohm), which continuously measured the voltage and sent output to the PC. The voltmeter was also kept inside the thermostated chamber at the same temperature as the electrochemical cell.

Inoculation. Electrochemical cell was inoculated with a suspension of supragingival and interdental plaque obtained from a healthy volunteer that did not have any titanium implants. The plaque was collected with a fresh 20 ul pipette tip by inserting the tip between the teeth and running the tip along the gumline. The plaque was resuspended in 1 ml of sterile medium and the entire volume was injected though one of the needle ports of the electrochemical cell (Fig 1).

#### Batch suspended cell cuture

Immediately after inoculation, filter-sterilized air was supplied to the reactor head space through one of the needle ports. The second needle port acted as gas exhaust, which was vented through a checkvalve followed by a hydraulic back-pressure valve (bubbler). The reactor system was closed and operated batch-wise, except for the supply of air.

Continuous reactor cultures. The flow regime was conducted with the version of the electrochemical cell having two inlet/outlet ports (Fig 1). Immediately after inoculation, one needle port was sealed, while the other was closed with a Millipore 0.22 um syringe filter. The upper outlet was constantly evacuated with a peristaltic pump, initially drawing air through the headspace. Inoculated microoganisms were allowed to settle in the system and adhere to exposed surfaces for 2 h, after which the delivery of fresh sterile medium supplemented with oxygen indicator resazurin (2 mg/l) was initiated through the lower inlet port. The spent medium and any suspended microbial cells left by way of the upper port. The inlet medium flow rate was set at 2.5 ml/min, with a reactor volume = 50 ml, this translated to a reactor fluid residence time of ~ 20.0 min. The reactor at this residence time was operated under predominantly “washout” conditions, thus minimizing the growth of suspended cells. Under these conditions the only bacteria in the reactor would be attached to a surface.

### Inductively Coupled Mass Spectrometry

Samples from the clinic were transported to the laboratory in screw-cap tubes. Since the tubes were incompatible with microwave digestion, the samples were quantitatively transferred to digestion vessels (50 mL polypropylene centrifuge tubes) with four 1 ml rinses of digestion solution. The digestion solution was 50:50 (V/V) concentrated nitric acid (Fisher, trace-metal grade): deionized (DI) water (Barnstead Nanopure, ≥ 18 MOhm∙cm^-1^) with a trace amount of HF (BDH, Aristar plus) and 10 ppm Tb (BDH) as recovery standard. The transferred sample was brought to 5 ml with the digestion solution. Open vessel microwave (Mars Xpress, CEM) digestion was used (power 800W, 100%, ramp 15 min to 100°C, hold for 45 min). After the digestion, samples were brought to 25 ml with DI water. Analysis for ^47^Ti was conducted by inductively coupled mass spectrometry (ICPMS; Agilent 7500CE with ASX-510 autosampler; 1500W power, no-gas mode) using internal standard (^45^Sc) calibration. In order to eliminate polyatomic interferences [21], the instrument was run in He mode; the instrument detection limit was 0.01 ng/mL. The calibration standards (0.01–100 ppb) were at the same final acid concentration as the samples and were prepared from single element commercial standards (Ultra Scientific; certified reference material) with a check calibration standard prepared from an alternate lot or vendor (BDH). Results were corrected for process blank values.

### DNA Sequencing

DNA from the plaque and *in vitro* experiments was isolated using Chelex-100™ (Bio-Rad, USA), a styrene divinylbenzene copolymer containing paired iminodiacetate ions, which act as chelating groups in binding polyvalent metal ions [22]. A 150 ul aliquot of suspended plaque was placed into a tube containing 10 mg Chelex 100 followed by addition of 50 ul of 120 mM Tris HCl pH 8.0 followed by addition of 10 ul of 10 mg/mL proteinase K. Proteinase K was dissolved in 30 mM Tris HCl, pH 8.0. The mix was incubated at 55°C for 30 min followed by vortexing and incubation in a boiling-water bath for 8 min. Upon removal from the boiling water bath, the tubes were centrifuged at 10,000–15,000×g for 3 min and the supernatant was transferred to a clean 1.5 ml microcentrifuge tube. Prokaryotic 16S rRNA genes were amplified using universal primers (27F and 1392R) using the GemTaq kit from MGQuest (USA) (Cat# EP012). The PCR program involved a pre-amplification step of 10 cycles with annealing temperature of 56°C followed by 20 amplification cycles with annealing temperature 58°C. In each cycle, elongation time was 1 min 10 s, at 72°C. PCR was finalized by extended elongation for 5 min. PCR products were purified with DNA Clean & Concentrator columns (Zymo Research, USA) and quantified using the NanoDrop (Agilent, USA).

Each purified PCR product, 1 ug, was labeled with a Multiplex Identifier (MID) during the Roche Rapid Library preparation step. Six MID-tagged sequences, representing six samples, were combined in equimolar concentrations and subjected to emPCR and DNA sequencing protocols as specified by the manufacturer’s recommendations for the Roche 454 Jr instrument.

### DNA Data analysis

The obtained sequences were separated out by their respective Multiplex Identifier (MID) and uploaded to the MG-RAST web server [23]. The MG-RAST pipeline assessed the quality of sequences, removed short sequences (multiplication of standard deviation of length cutoff of 2.0) and removed sequences with ambiguous base pairs (non-ACGT; maximum allowed number of ambiguous base pair was set to 5). The pipeline annotated the sequences and allowed the integration of the data with previous metagenomic and genomic samples. The M5RNA database was used as annotation source, with minimum sequence identity of 97%, maximum e-value cutoff at 10^−5^, and minimum sequence length of 100 bases. PCA was conducted in MG-RAST.

## Results

### Titanium corrosion *in vivo*

**Fig 2:**
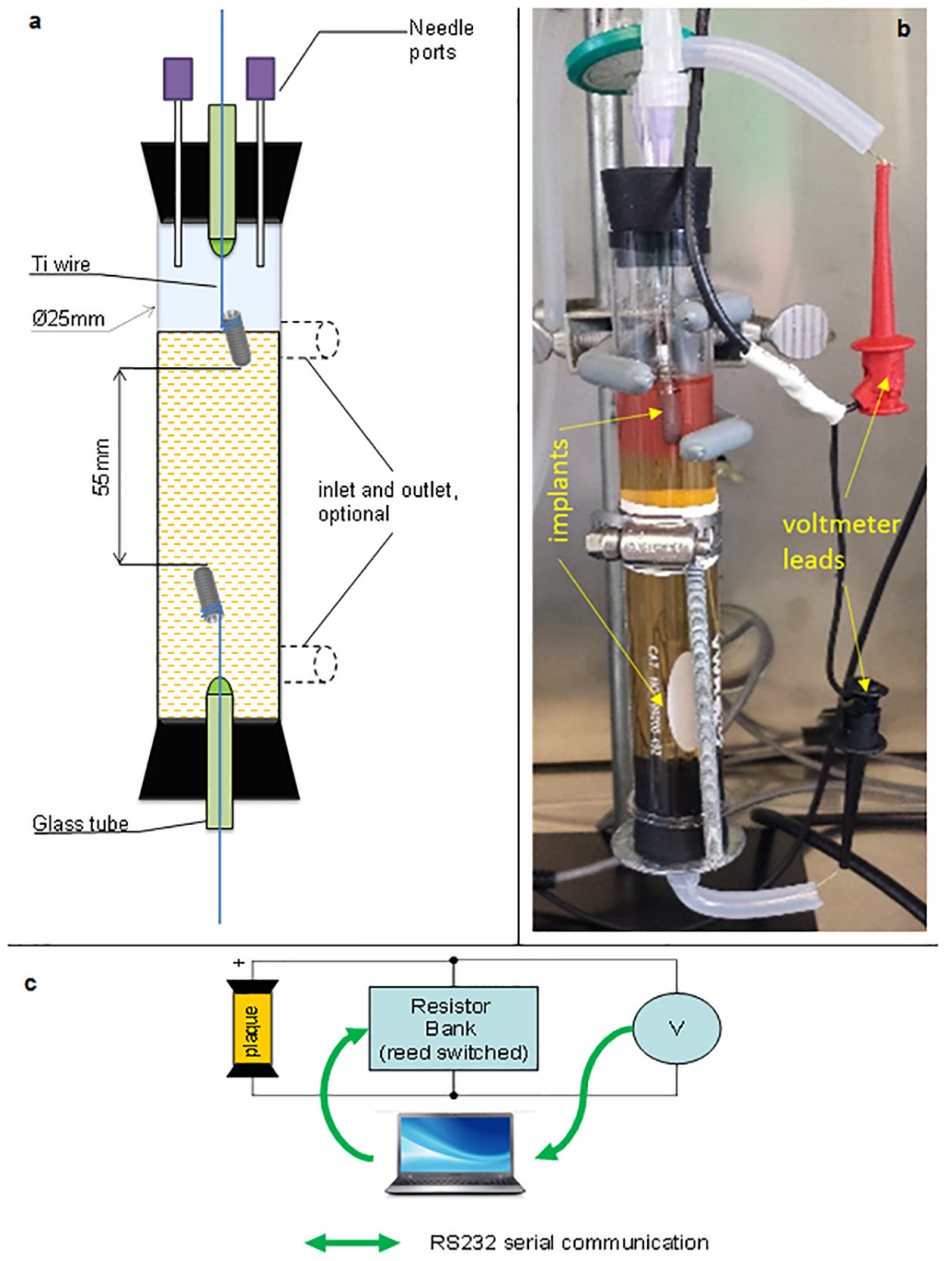
Distribution of measured titanium quantities (log10 scale) recovered from patient samples for the 28 sites. There were additional 26 sites for which the quantities of titanium fell below the detection limit.

In order to assess natural variability of titanium quantities leached into the patients’ plaque, we collected 54 samples from 34 patients. Titanium was detected in 28 plaque samples corresponding to 16 patients. The distribution of the measured quantities is shown on Fig 2. Sites with peri-implantitis tended to have on average more titanium than the healthy or mucositis sites. The difference between the means on log-scale was 0.44, (0.82 versus 0.38, respectively, p = 0.08). After regression adjustment for patient age and gender as well as the amount of DNA obtained at each site, the difference between groups was reduced to 0.29 and became less significant (p = 0.28). Note, the amount of DNA was used as a proxy for the amount of collected subgingival plaque (since plaque is composed of epithelial and microbial cells). Comparisons of titanium levels were performed using Generalized Estimating Equations to account for clustering of sites within patients.

### Titanium corrosion *in vitro*

An electrochemical cell was designed that simulates an implant in a mouth (Fig 1). To investigate the apposition of metabolite concentration gradients over the metallic implant, a symmetric membrane-less fuel cell was developed. The metabolite concentration gradients were spontaneously generated between the oxygenated and anaerobic areas at the top and at the bottom of the cell, respectively. The placement of two identical titanium implants in the oxygenated and anaerobic zones of the cell, as cathodic and anodic counterparts of the operational fuel cell, allowed controllable investigation of electrochemical processes of implants in the mouth. Essentially, the electrochemical cell represents a scaled-up implant with its body cut in half and an electrical load as well as a voltmeter inserted in between the two halves.

**Fig 3:**
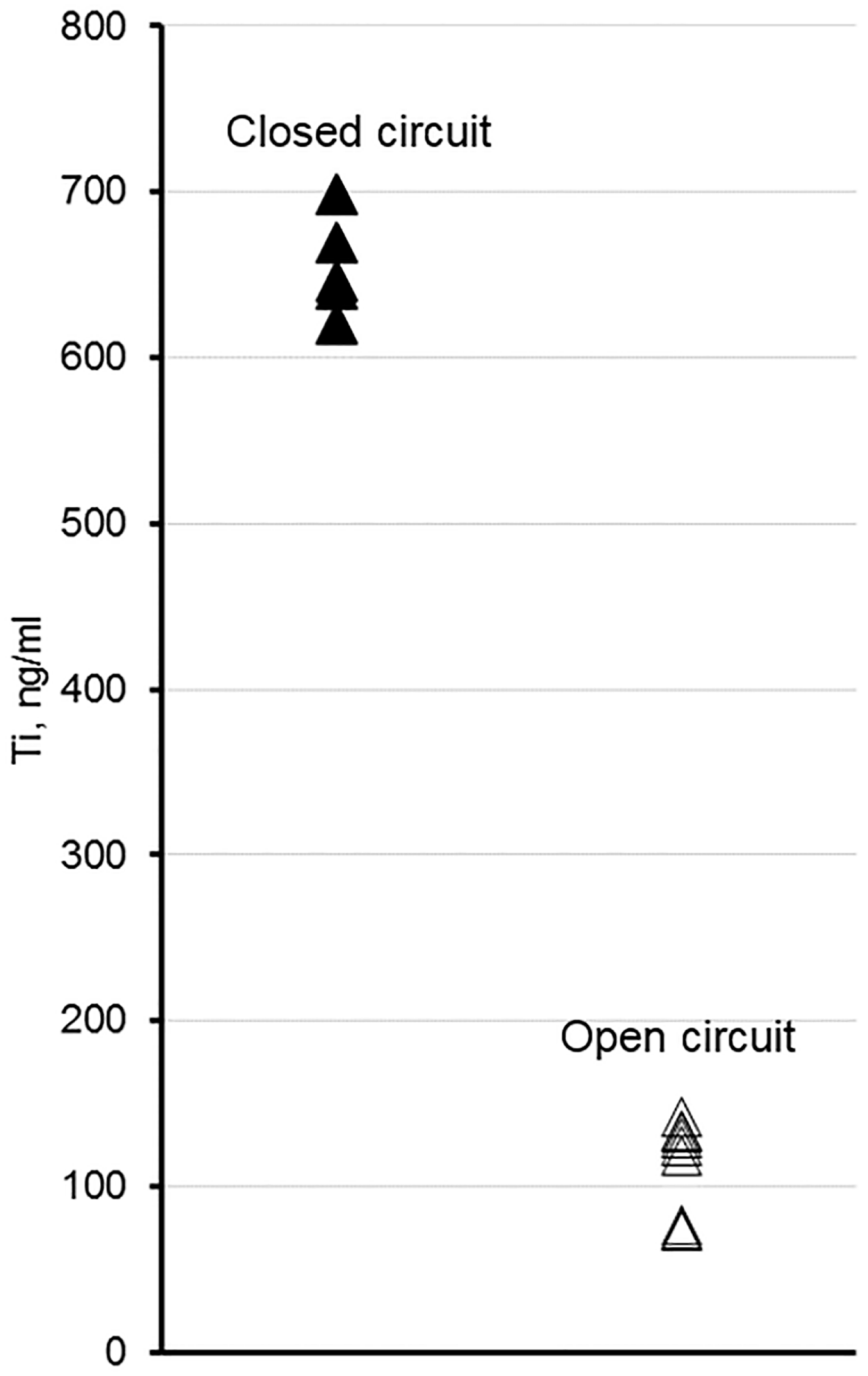
Titanium concentrations measured in the electrochemical cell after 90 h of incubation depending on the circuit regime.

In the mouth, the body of the implant is intact and electrical conduction occurs without interruption. In order to simulate this situation in the laboratory and at the same time be able to monitor electrochemical (incl. biocorrosion) processes, the electrochemical cell was operated in the “closed-circuit” regime (i.e., kinetic regime). Specifically, in the repeated fashion, the cell terminals were loaded with ~0 kOhms for 60 min followed by a 10 min 100 kOhm load. The alternated load was repeated for about 80 h. The 100 kOhm load enabled measurements by the voltmeter. The 10 min interval was chosen based on preliminary experiments that indicated the voltage across the resistor stabilized within 10 min. Essentially, the electrochemical cell was shorted for 86% of the time, which we believe is a close approximation to the situation in the mouth. The closed-circuit regime experiment was conducted in triplicate. The other test was conducted in an “open-circuit” regime (i.e., thermodynamic regime), which simulated a potential new design of an implant where electrical conduction could be interrupted. The open-circuit regime experiment was conducted in triplicate. Fig 3 shows comparison between the quantities of corroded titanium that leached into the growth media for open versus closed circuit regimes. Note, the measurements of titanium quantities leached into the electrochemical cell were additionally replicated to ensure reproducibility of the ICPMS instrument. In total, there were 5 measurements for the closed-circuit and 9 for open-circuit regimes. Apparently, the open circuit regime resulted in four to five-fold reductions in titanium corrosion.

**Fig 4:**
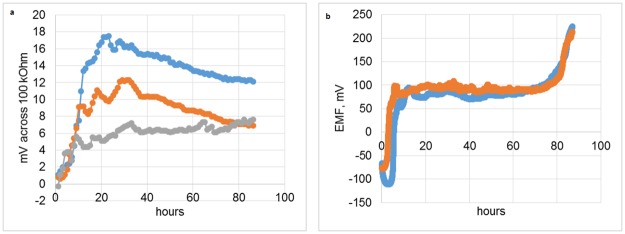
Voltage measurements from two circuit regimes. a, closed-circuit regime, i.e., 60 min ~0 kOhm, 10 min 100 kOhm load; a value at the end of the 10 min interval is shown; each replicated measurement run is distinctly colored. b, open circuit, the electrochemical cell is directly connected to the voltmeter, no load; sampling is performed every minute; each replicated measurement run is distinctly colored.

To determine if corrosion in the *in vitro* model was due to microorganisms, the metal, or a combination of the two, we investigated electrochemical dynamics of the cell under closed- and open- circuit regimes. The results show that electrochemical activity was similar at the beginning of the process (Fig 4), presumably due to microbial growth as noted by increased turbidity (Fig 4), but after 20 h, the activity became highly variable and varied by experiment as shown by the differently colored profiles.

**Fig 5:**
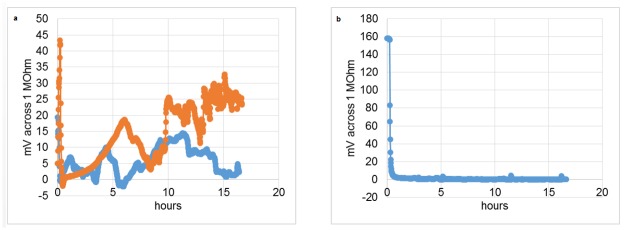
Electrochemical cell in a flow-through regime. A flow of media was established at 0.05 volumes/min through the inoculated (a, two replicates shown) and sterile (b) electrochemical cells.

The observed electrochemical activity could also have been generated by oxygen depletion in the lower part of the electrochemical cell (Fig 1). The oxygen depletion was evident by the redox indicator, which was red only in the upper part of the cell. The oxygen depletion could be due to reduction of oxygen by the media, consumption by the microorganisms, or both. To test if microorganisms were capable of sustaining an electrochemical gradient, a flow-through regime was investigated. Results show that microorganisms in the biofilm were responsible for sustained electrochemical activity as shown by the difference in electrochemical activities of the inoculated versus the sterile cell (Fig 5).

### Microbial composition *in vitro* and *in vivo*

**Fig 6:**
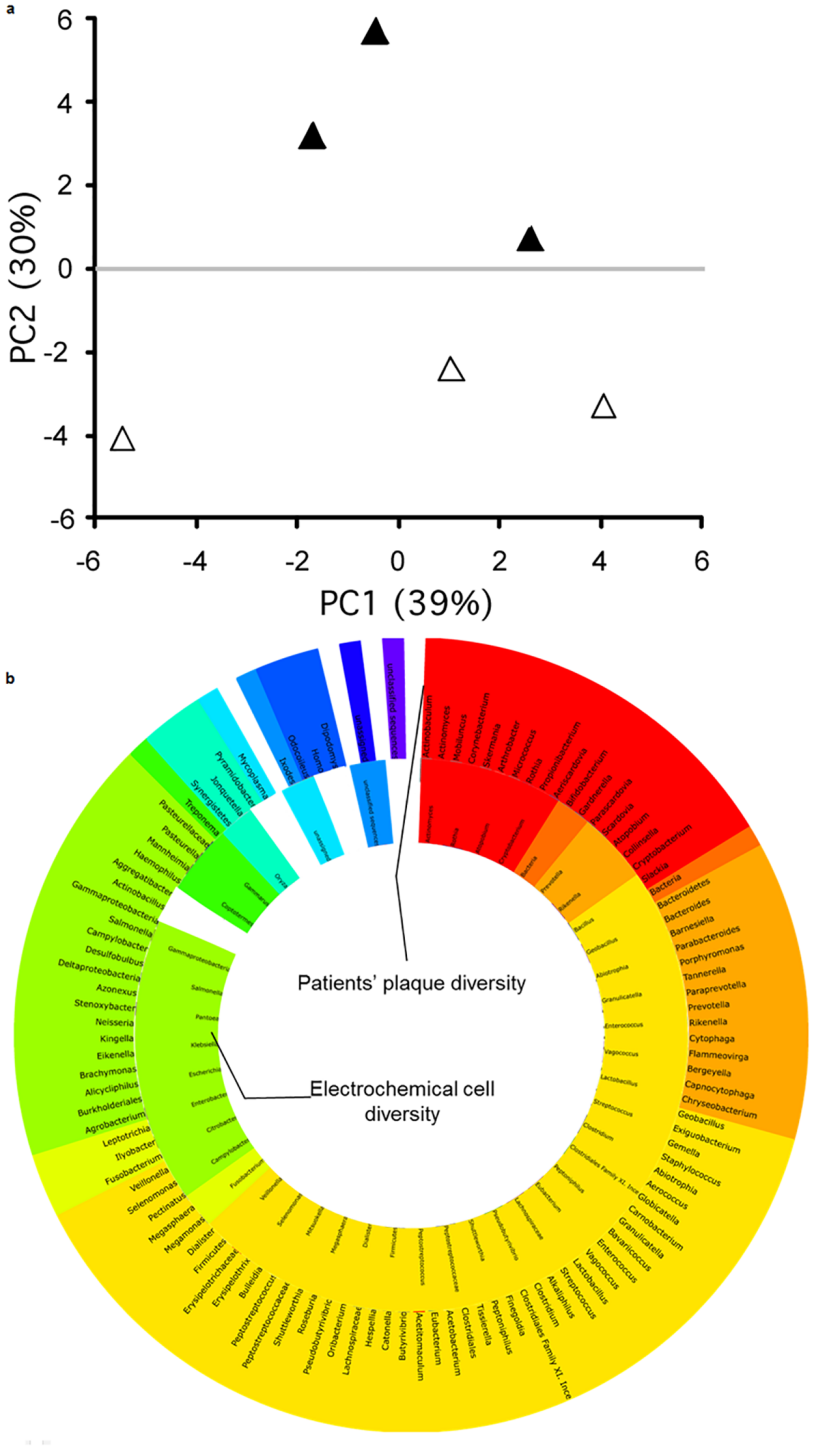
a, ordination plot based on principal coordinate analysis of the microbiomes obtained from the electrochemical cell operating in the open circuit (open triangles) and closed circuit (solid triangles) regimes. The ordination accounts for 68.5% of variability. B, comparison of microbial compositions for samples obtained from thirteen patients and electrochemical cell. Color indicates phylum level.

Microbial community composition in the electrochemical cell under open and closed circuits were distinctly different (Fig 6). The ordination plot accounted for 69% of the variability in two axes. Examination of the microbial community composition of closed- and open-circuit regimes revealed differences in the presence of several microbial genera (S1 Fig).

Comparison of the microbial community composition and diversity of the electrochemical cell to that of the dental plaque from thirteen patients revealed some overlap in bacterial species. Specifically, 33 bacterial genera were found in both the plaque samples and the electrochemical cells. Eleven genera were found in the plaque samples only, while 72 genera were found in electrochemical cell only.

## Discussion

Two important findings of our study are: corrosion of dental implants occurs in at least half of the studied patients and the extent of corrosion might be reduced four to five-fold by interrupting the electrical current flow through the implant. Although titanium implant corrosion has been reported before and it has been suggested that corrosion could be one of the causes for implant failure [7, 12, 24], there is a paucity of information on the actual distribution of titanium quantities leached into the implant milieu. Our study fills the void by showing that, contrary to the observation by Olmedo et al. [12], there is no strong association found between health, peri-implantitis severity and quantities of leached titanium. One of the reasons for the discrepancy could be that Olmedo et al. [12] collected samples from the surface of the gum far away from the actual implant and all the samples were pooled based on the diagnosis of peri-implantitis. The pooled samples were measured once; hence, no statistical assessment was possible. In addition, although precautions were taken not to scuff the implant, it is possible that during our sample collection procedure the implant was grazed by the curette and the scraped-off material could have contributed to the variability. It is important to note that in our study, we measured a combined effect of electrochemical / electromicrobiological corrosion and mechanical wear onto the quantities of leached titanium *in vivo*.

An indirect effect of the titanium corrosion products on alveolar bone resorption has been recently demonstrated in the rat model [25]. Specifically, titanium ions and lipopolysaccharide from *P*. *gingivalis* synergistically act upon the CCL2-RANKL-OPG system, which is known to be associated with inflammation and bone resorption [26, 27]. In addition, it was found that a direct stimulation of macrophages by titanium oxide results in secretion of pro-inflammatory cytokines TNF-a and IL-1b, which in turn, are secreted in patients with failed implants [28].

Because titanium is the best material for dental implants due to its superior osseointegration capability compared to other materials, it would be interesting to investigate possible ways to reduce its corrosion effects. Titanium dental implants could be considered as an electrochemical system, whereby one zone of the implant acts as a biocathode, while the other acts as a bioanode covered by a biofilm. This fact led us to question: *do biofilms require destruction of the biocathode and the bioanode to thrive*? While it is known that some biofilms do not destroy the material on which they grow [29], there are numerous reports on microbiologically influenced corrosion of metals. The review by Li et al. [30] outlines a consensus theory on microbiologically-influenced corrosion and shows potential ways to mitigate it. In a clinical dental implant setting, however, none of these corrosion mitigation strategies are realistic—and neither is it realistic to completely remove biofilm from a dental implant.

In this study, we investigated a strategy to interrupt electrical current between the cathodic and anodic zones of the implant that could potentially result in disruption of the corrosive biofilm. The reason this is important is because a disruption of the electrical current resulted in a reduction of corrosion. Hence, one could design a new implant material, such a ceramic sintered with titanium powder, which is exposed to the surface for osseointegration. Experiments on creating and evaluating such material are underway in our lab.

With our new understanding that electrons can flow between cathodic and anodic zones of an implant, we hypothesized the existence of a ‘vicious cycle’. The cycle consists of a corrosive biofilm that uses titanium implant for its growth. Titanium electroconductivity supports microorganisms that corrode titanium directly or indirectly. In order to test the hypothesis, we have designed a reactor that closely mimics natural implant environments and allows intervention between the cathodic and anodic zones of the implant (Fig 1). Our reactor was different from those used in other studies for two reasons: our reactor features real dental implants with the surface activated for osseointegration and was inoculated with native microbiota from the human mouth. Other studies used one or two microorganisms in a biofilm [10, 11], and none used actual dental implants [31, 32].

The reactor used in our study essentially works as a microbial fuel cell and its output indirectly reflected upon the dynamics of microbial population changes (Fig 4). The open-circuit regime seems to show nearly identical replicated voltage-time dependencies, while for the closed circuit there were considerable differences in the amplitude of the dependencies as well as the shape. The explanation for the discrepancy is that the open circuit regime measurements determine the thermodynamic potential difference between cathode and anode. This thermodynamic potential difference only reflects the energy differences between the cathode and anode, however, no actual reaction is occurring at any appreciable rate. On the other hand, the closed-circuit voltage measurements are proportional to the current generated by the system, which in turn directly reflects upon the electrochemical transformations occurring in the system. The difference between the extents of corrosion for the closed- and open-circuit regimes was striking (Fig 3). The residual corrosion observed in the open-circuit regime is likely due to the somewhat corrosive metabolites (e.g., inorganic and organic acids) produced by the microorganisms; however, the strong corrosive effect of the biofilms observed in the closed-circuit regime was not observed. This finding indicates that a path towards the reduction of the dental implant corrosion indeed lies through interrupting the current between the implant’s cathodic and anodic zones. Of note, the circuit regime apparently altered the microbial communities (Fig 6), which gives credence to the ‘vicious cycle’ hypothesis.

One could argue that bacteria are not forming an electroactive biofilm and the observed voltage dynamics is solely determined by an oxygen gradient, i.e., the oxygen is simply consumed by the reductive media and bacteria in the anode zone, while cathode zone is perfused with oxygen by the peristaltic pump. In order to test if the biofilm was electroactive, we performed a flow-through experiment with fully oxygenated media (as observed by resazurin). At the volumetric dilution rate of 0.05 min^-1^, the retention time in the reactor was ~20 min. It should be noted that the normal flow rate of the gingival crevicular fluid (CGF) in a healthy pocket is 0.5 volumes per min [33]. The normal CGF flow rate as well as the one tested in our reactor renders the microbial growth to a predominant washout. It can be shown that the washout regime occurs when the volumetric dilution rate exceeds the maximum growth rate of the fastest growing microorganisms given the abundance of the substrate in the influx. Despite the washout, a thick growth of biofilm was observed as well as the electrochemical activity measured (Fig 5) indicating that the biofilm was indeed electroactive.

There are a number of descriptive studies covering microbial diversity of peri-implantitis and periodontitis (e.g. [5, 34, 35]). Our focus was not to repeat such efforts, however to establish adequacy of the *in vitro* model, a cursory comparison of *in vivo* and *in vitro* microbial diversities was conducted. One could argue that *in vitro* conditions and chosen growth medium could have selected only a handful of microorganisms in the electrochemical cell. Microorganisms from the plaque of thirteen patients with and without peri-implantitis were sequenced and names of the microbial genera were combined into one list. Another list was composed from microbial genera obtained from sequencing microbiota of the electrochemical cell. Fig 6 shows comparison of the microbial genera recovered. It is interesting to note that some microorganisms found in the cells that were not found in the plaque samples. The reason for this discrepancy could be due to the fact that sequencing was non-exhaustive. In any case, electrochemical cell shows extensive microbial diversity, which makes it an adequate model for studying implants. Interestingly, the relative proportion of genera detected by the 16S rRNA gene sequencing for the open and closed circuit regimes was clearly different between the regimes (S1 Fig). For example, there are more *Escherichia*, *Pantoea*, *Eubacterium*, *Bacillus*, *Clostridium*, *Megasphaera*, *Pseudobutyrvibrio*, *Peptostreptococcus*, and *Firmicutes* in biofilms associated the open regime than the closed regime. In contrast, there are more *Klebsiella*, *Citrobacter*, *Salmonella*, *Rothia*, *Mitsuokella*, *Vagococcus*, *Geobacillus*, *Rikenella*, and *gammaproteobacteria* in biofilms associated the closed regime than the open regime. Since most of these bacteria are capable of producing acids and therefore contribute to corrosion by changing the pH locally, it is difficult to make further interpretations without a detailed gene expression analysis.

Titanium is currently considered the gold standard for dental implants due to its excellent osseointegration rates, in addition to the strength and flexibility of the material. While many other materials have been explored as substitutes, they have had problems with osseointegration, were too weak, or were not flexible enough (Table 1). Thus, titanium continues to be the most widely used in dental implantology. However, we now know that titanium corrosion occurs due to a bioelectrochemical reaction, leading us to the conclusion that a new type of dental implant should be created in order to interrupt the generation of electricity that occurs with traditional titanium implants. As seen in Table 1, aluminum oxide ceramic implants were not strong enough when used alone; however, implant failure was not associated with peri-implantitis or bone disintegration. This leads us to believe that this material eliminated peri-implantitis and bone disintegration problems due to its characteristic of non-conductivity. We hypothesize that a new type of dental implant could be created using a combination of titanium, for strength and osseointegration, and aluminum oxide ceramic, in order to eliminate any bioelectrochemical reactions.

**Table 1:** Materials used in dental implantology

Material	Name	Success/ Survival	Notes	Ref.
iridium-platinum alloy	Greenfield’s implant system	Unknown	First successful implant	[37]
cobalt-chromium-molybdenum alloy	Vitallium	Unknown	First long-term successful implant; the osseointegration is worse than Ti	[38], [39]
vitreous carbon	Vitrident	25–35% after 5 years survival rate	Not strong enough and low osseointegration rates; withdrawn from market	[40], [41], [42]
LTI pyrolytic carbon	LTI pyrolytic carbon implant	60% after 5 years success rate	Very low success rate	[43]
Single-crystal alumina ceramic	single crystal sapphire implants	68.66% after 14 years survival rate	Low success rates; inferior to Ti	[44]
polymethyl-methacrylate	PMMA	Unknown	Issues including cervical infection, loosening, and deterioration	[45]
polycrystalline alumina ceramic	Tübingen implant	92.5% success rate	Low fracture resistance, withdrawn from market; implant failure was not associated with peri-implantitis or bone disintegration	[46], [47], [48], [49]
SiO_2_, Ca, Na_2_O, H, and P coating	Bioactive glass/Bioglass coating	Uncertain	Still in use	[50], [51], [52], [53]
zirconium dioxide	Zirconia implant	74–98% after 1–5 years survival rate	Still in use	[49], [54]

Electrical stimulation has been found to enhance bone growth and healing, but Gittens et al. [36] also found that bone cells are sensitive to the currents produced during corrosion events of metallic implants. This is because compared to intact surrounding tissue, bone has a neutral potential when mature, negative during growth, and positive during resorption. We suggest that a new electrotherapy could be developed to promote bone healing and to influence the growth of microbial biofilms in patients with peri-implantitis.

## Conclusion

In conclusion, our study demonstrated that titanium corrosion occurs in patients with dental implants. The quantities of released titanium due to corrosion are quite variable within the studied population. An *in vitro* model was established opening up numerous possibilities for research on mechanisms and ways of mitigation of the dental implant biocorrosion. The *in vitro* model also demonstrated that microorganisms are capable of sustaining a chemical gradient that leads to spontaneous generation of electricity and corrosion of titanium. Interruption of the electrical current resulted in a reduction of corrosion. These findings suggest the need for a new type of dental implant: a combination of titanium metal parts or particles (for osseointegration) interlaid with electrically nonconductive material.

## Supporting Information

S1 Fig Differences between microbial compositions on the genus level for open- and closed-circuit regimes. Color indicates microbial phyla.
(TIF)

S2 Fig Electrical schematic of the interface circuit. X1 connects to the voltmeter, X2 to the PC, SV2 to the resistor bank.
(TIF)
