# Supplementary figures and images for: Interruption of Electrical Conductivity of Titanium Dental Implants Suggests a Path Towards Elimination Of Corrosion
Source: PLoS One. 2015 Oct 13;10(10):e0140393. doi: 10.1371/journal.pone.0140393 (PMC4604158; doi:10.1371/journal.pone.0140393)

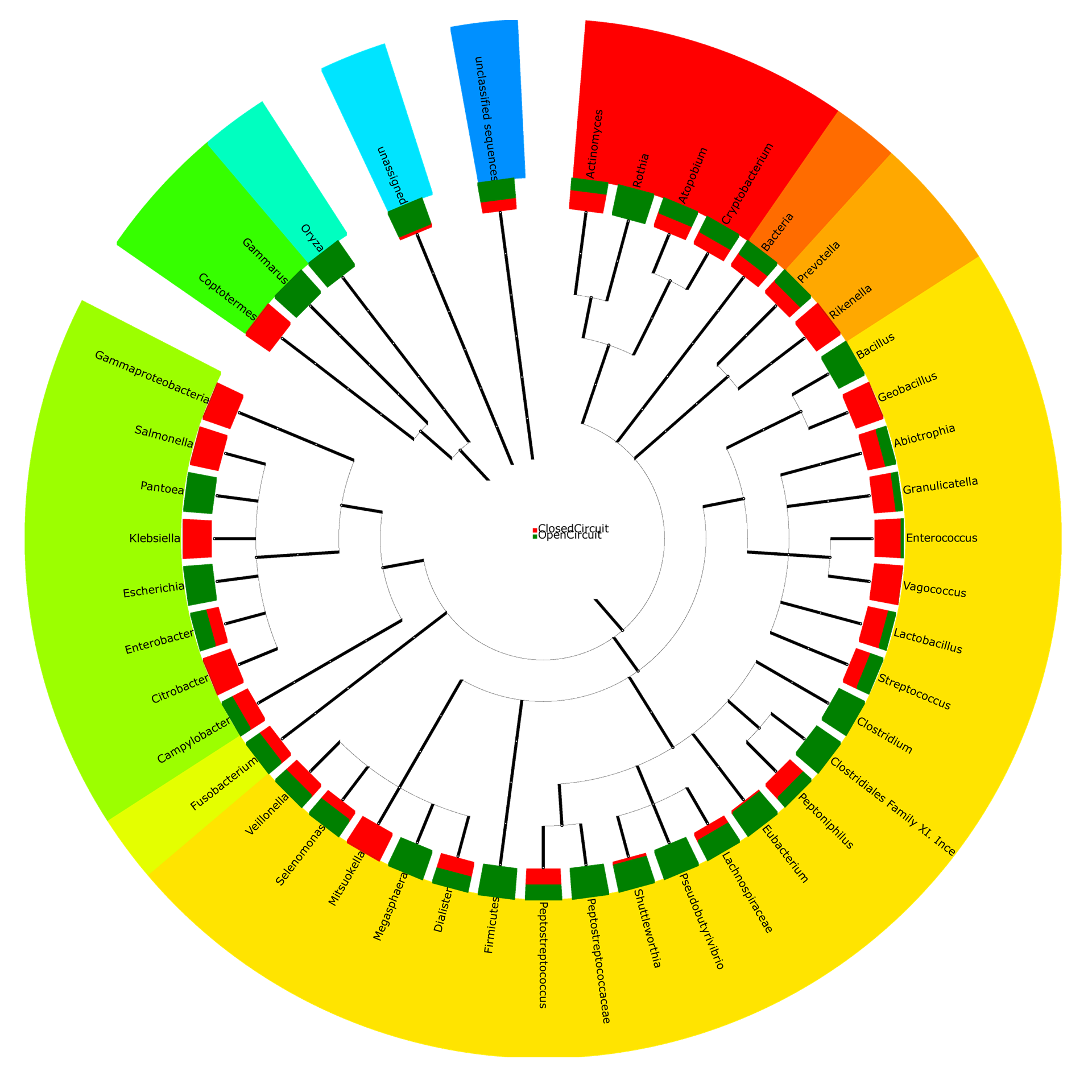

Supplement: S1 Fig — Color indicates microbial phyla. (TIF) [file pone.0140393.s001.tif]

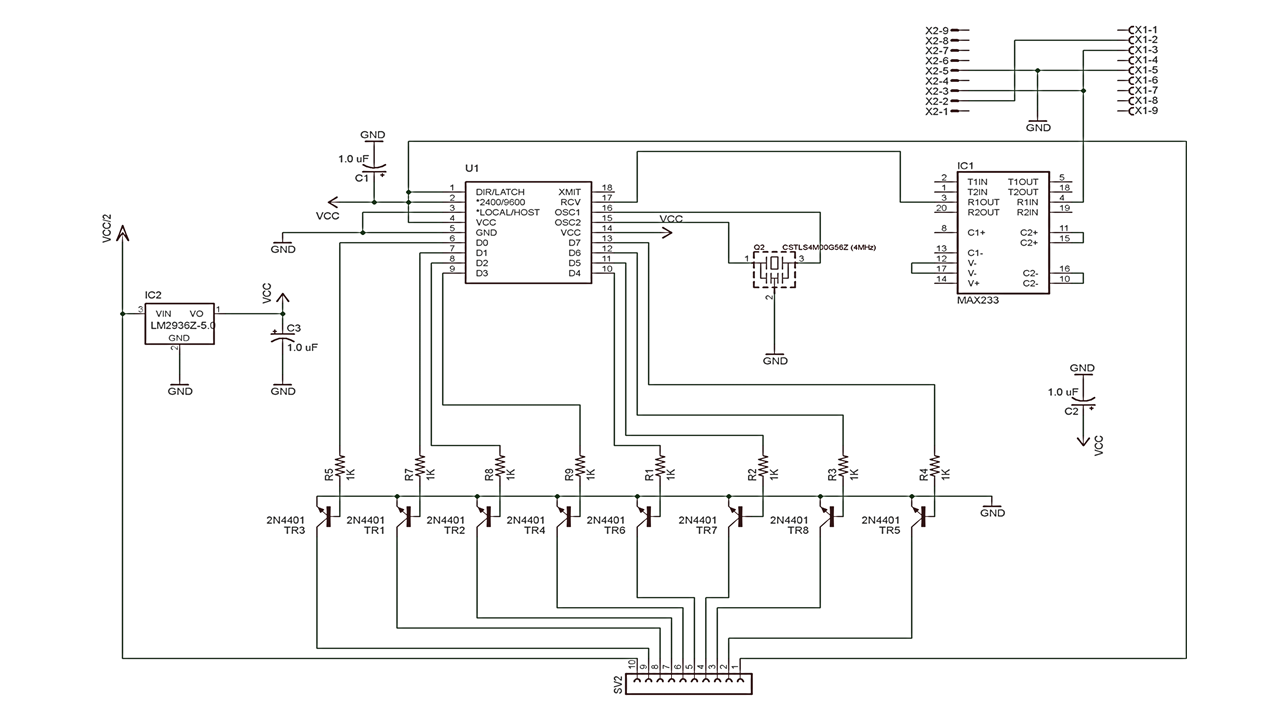

Supplement: S2 Fig — X1 connects to the voltmeter, X2 to the PC, SV2 to the resistor bank. (TIF) [file pone.0140393.s002.tif]
